# Efficient use of unlabeled data for protein sequence classification: a comparative study

**DOI:** 10.1186/1471-2105-10-S4-S2

**Published:** 2009-04-29

**Authors:** Pavel Kuksa, Pai-Hsi Huang, Vladimir Pavlovic

**Affiliations:** 1 Department of Computer Science, Rutgers University, Piscataway, NJ, 08854, USA

## Abstract

**Background:**

Recent studies in computational primary protein sequence analysis have leveraged the power of unlabeled data. For example, predictive models based on string kernels trained on sequences known to belong to particular folds or superfamilies, the so-called labeled data set, can attain significantly improved accuracy if this data is supplemented with protein sequences that lack any class tags–the unlabeled data. In this study, we present a principled and biologically motivated computational framework that more effectively exploits the unlabeled data by only using the sequence regions that are more likely to be biologically relevant for better prediction accuracy. As overly-represented sequences in large uncurated databases may bias the estimation of computational models that rely on unlabeled data, we also propose a method to remove this bias and improve performance of the resulting classifiers.

**Results:**

Combined with state-of-the-art string kernels, our proposed computational framework achieves very accurate semi-supervised protein remote fold and homology detection on three large unlabeled databases. It outperforms current state-of-the-art methods and exhibits significant reduction in running time.

**Conclusion:**

The unlabeled sequences used under the semi-supervised setting resemble the unpolished gemstones; when used as-is, they may carry unnecessary features and hence compromise the classification accuracy but once cut and polished, they improve the accuracy of the classifiers considerably.

## Introduction

Classification of proteins into structural or functional classes is one of the fundamental problems in computational biology. With the advent of large-scale sequencing techniques, experimental elucidation of an unknown function of the protein sequence becomes an expensive and tedious task. Currently, there are more than 61 million DNA sequences in GenBank [1], and approximately 349,480 annotated and 5.3 million unannotated sequences in UNIPROT [2], making development of computational aids for sequence annotation a critical and timely task. In this work we address the problem of remote fold and homology prediction using only the primary sequence information. While additional sources of information, such as the secondary or tertiary structure, may lessen the burden of establishing functional or structural similarity, they may often be unavailable or difficult to acquire for new putative proteins. Even when present, such information is only available on a very small group of protein sequences and absent on larger uncurated sequence databases.

We focus on performing *remote *fold and homology detection with kernel-based methods [3] that use sequence information only under the *discriminative *learning setting. The discriminative learning setting captures the *differences *among classes (e.g. folds and superfamilies). Previous studies [4,5] show that the discriminative models have better distinction power over the generative models [6], which focus on capturing *shared characteristics within *classes.

Remote fold and homology detection problems are typically characterized by few *positive training *sequences (e.g. sequences from the same superfamily) accompanied by a large number of negative training examples. Lack of positive training examples may lead to sub-optimal classifier performance, therefore making training set expansion necessary. However, enlarging the training set by experimentally labeling the sequences is costly, leading to the need to leverage available *unlabeled data *to refine the decision boundary. The profile kernel [7] and the mismatch neighborhood kernel [8] both use unlabeled data sets and show significant improvements over the sequence classifiers trained under the supervised (labeled data only) setting. In this study, we propose a systematic and biologically motivated approach that more efficiently uses the unlabeled data and further develops the crucial aspects of neighborhood and profile kernel methods. The proposed framework, the *region-based neighborhood method *(Section 'Extracting relevant information from the unlabeled sequence database'), utilizes the unlabeled sequences to construct an accurate classifier by focusing on the significantly similar sequence regions that are more likely to be biologically relevant. As overly-represented sequences may lead to performance degradation by biasing kernel estimations based on unlabeled data, we propose an effective method (Section 'Clustered Neighborhood Kernels') that improves performance of the resulting classifiers under the semi-supervised learning setting. Our experimental results (Section 'Experiments') show that the framework we propose yields significantly better performance compared to the state-of-the methods and also demonstrates significantly reduced running times on large unlabeled datasets.

## Background

In this section, we briefly review previously published state-of-the-art methods for protein homology detection and fold recognition. We denote the alphabet set of the 20 amino acids as Σ in the whole study.

### The spectrum kernel family

The spectrum kernel methods [5,9] rely on fixed-length representations or features Φ(*X*) of arbitrary long sequences *X *modeled as the *spectra *(|Σ|^*k*^-dimensional histogram of counts) of short substrings (*k*-mers) contained in *X*. These features are subsequently used to define a measure of similarity, or kernel, *K*(*X*, *Y*) = Φ(*X*)^*T*^Φ(*Y*) between sequences *X*, *Y*.

Given a sequence *X*, the *mismatch(k*, *m) *kernel [5] induces the following |Σ|^*k*^-dimensional representation for *X*:

(1) $$\Phi^{k,m}(X)={\left(\sum_{\alpha \in X}I_{m}(\alpha ,\gamma )\right)}_{\gamma \in \Sigma^{k}},$$

where *I*_*m*_(*α*, *γ*) = 1 if *α *∈ *N*(*γ*, *m*) and *N*(*γ*, *m*) denotes the set of contiguous substrings of length *k *that differ from *γ *in at most *m *positions.

Under the mismatch(*k*, *m*) representation, the notion of similarity is established based on *inexact matching *of the *observed *sequences. In contrast, the profile [7,10] kernel, proposed by Kuang et al., establishes the notion of similarity based on a probabilistic model (profile). Given a sequence *X *and its corresponding profile [11], the |Σ|^*k*^-dimensional profile(*k*, *σ*) representation is:

(2) $$\Phi^{profile(k,\sigma )}(X)={\left(\sum_{i=1\cdots (T_{P_{X}}-k+1)}I(P_{X}(i,\gamma )<\sigma )\right)}_{\gamma \in \Sigma^{k}},$$

where *σ *is a pre-defined threshold, $T_{P_{X}}$ denotes the length of the profile and *P*_*X*_(*i*, *γ*) the cost of *locally *aligning the *k*-mer *γ *to the *k*-length segment starting at the *i*^*th *^position of *P*_*X*_.

Explicit inclusion of the amino acid substitution process and leveraging the power of the unlabeled data allow both the mismatch and profile kernels to demonstrate state-of-the-art performance under both supervised and semi-supervised settings [8,10,12]. Under the semi-supervised setting, the profile kernel uses the unlabeled sequences to construct a profile for inexact string matching whereas mismatch kernels take advantage of the *sequence neighborhood smoothing *technique presented in Section 'The sequence neighborhood kernel'.

### The sparse spatial sample features

Similar to the mismatch kernel, the *sparse spatial sample kernels *(SSSK) [13] also directly extract string features from the *observed *sequences. The induced features explicitly model mutation, insertion and deletion by sampling the sequences at different resolutions. The three parameters for the kernels are the sample size *k*, the number of samples *t*, and the maximum allowed distances between two neighboring samples *d*. The kernel has the following form:

(3) $$\begin{array}{l} K^{\left(t,k,d\right)}(X,Y)=\\ \sum_{\begin{array}{c} \left(a_{1},d_{1},...,d_{t-1},a_{t}\right)\\ a_{i}\in \Sigma^{k},0\leq d_{i}<d \end{array}}\begin{array}{c} C(a_{1},d_{1},\cdots ,a_{t-1},d_{t-1},a_{t}|X).\\ C(a_{1},d_{1},\cdots ,a_{t-1},d_{t-1},a_{t}|Y) \end{array}, \end{array}$$

**Figure 1:**
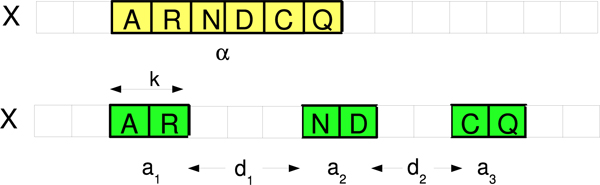
**Contiguous *k*-mer feature a of a traditional spectrum feature (top) contrasted with the sparse spatial samples (bottom)**.

**Figure 2:**
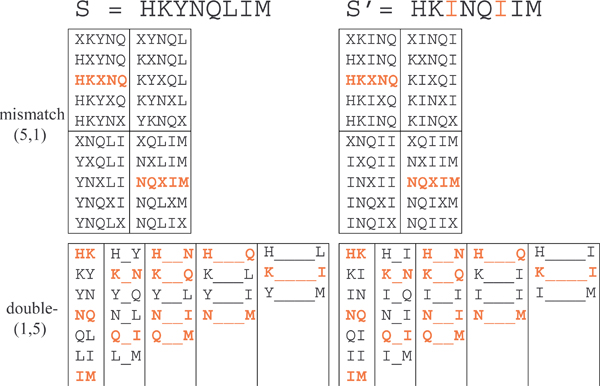
**Spectrum (*k*-mer) features vs. spatial sample features**.

Where *C*(*a*_1_, *d*_1_, ⋯, *a*_*t*-1_, *d*_*t*-1_, *a*_*t*_|*X*) denotes the number of times we observe substring $a_{1}\overset{d_{1}}{\underset{}{↔}}a_{2},\overset{d_{2}}{\underset{}{↔}},\cdots ,\overset{d_{t-1}}{\underset{}{↔}}a_{t}$ (*a*_1 _separated by *d*_1 _characters from *a*_2_, *a*_2 _separated by *d*_2 _characters from *a*_3_, etc.) in the sequence *X*. The crucial difference between the spatial and spectrum features is that the spectrum features consist of only *contiguous k*-mers, whereas the spatial sample features consist of a number of (*t*) *shorter k*-mers separated by some distance, (controlled by *d*), to directly model the complex biological processes. Such multi-resolutional sampling technique also captures *short-term dependencies *among the amino acid residues, or shorter *k*-mers, in the observed sequences. In Figure 1, we illustrate the differences between the spectrum and the spatial features. In the upper panel, we show a spectrum feature with *k *= 6 and in the lower panel, we show a spatial sample feature with *k *= 2, *t *= 3. Figure 2 further compares spectrum-like features with spatial sample features and shows mismatch(5,1) and double(1,5) feature sets for two strings, *S *and *S'*, that are similar but only moderately conserved (two mutations apart). More features are shared between *S *and *S' *under the spatial sample representation compared to the mismatch spectrum allowing to establish sequence similarity. Similar to the mismatch kernel, for the SSSK, semi-supervised learning can be accomplished using the *sequence neighborhood approach*. Kuksa et al. show in [13] that the SSSK outperform the state-of-the-art methods under the supervised setting and the semi-supervised setting on a small unlabeled data set.

### The sequence neighborhood kernel

The *sequence neighborhood *kernels take advantage of the unlabeled data using the process of neighborhood induced regularization. Let Φ^*orig*^(*X*) be the original representation of sequence *X*. Also, let *N*(*X*) denote the *sequence neighborhood *of *X *and *X *∈ *N*(*X*) (i.e. *N*(*X*) is the set of sequences neighboring (similar to) *X*; we will discuss how to construct *N*(*X*) in Sections 'Extracting relevant information from the unlabeled sequence database' and 'Experiments'). Weston et al. propose in [8] to re-represent the sequence *X *using its neighborhood set *N*(*X*) as

(4) $$\Phi^{new}(X)=\frac{1}{\left|N(X)\right|}\sum_{X^{\prime }\in N(X)}\Phi^{orig}(X^{\prime }).$$

Under the new representation, the kernel value between the two sequences *X *and *Y *becomes

(5) $$K^{nbhd}(X,Y)=\sum_{X^{\prime }\in N(X),Y^{\prime }\in N(Y)}\frac{K(X^{\prime },Y^{\prime })}{\left|N(X)||N(Y)\right|}.$$

Weston et al. in [8] and Kuksa et al. in [13] show that the discriminative power of the classifiers improve significantly once information regarding the neighborhood of each sequence is available.

### Proposed methods

In Section 'Extracting relevant information from the unlabeled sequence database', we first propose a new framework for extracting only relevant information from unlabeled data to improve efficiency and predictive accuracy under a semi-supervised learning setting. Next, we extend the proposed framework in Section 'Clustered Neighborhood Kernels' using clustering to improve computational complexity and reduce data redundancy, which, as we will show experimentally, further improves speed and accuracy of the classifiers.

### Extracting relevant information from the unlabeled sequence database

**Figure 3:**
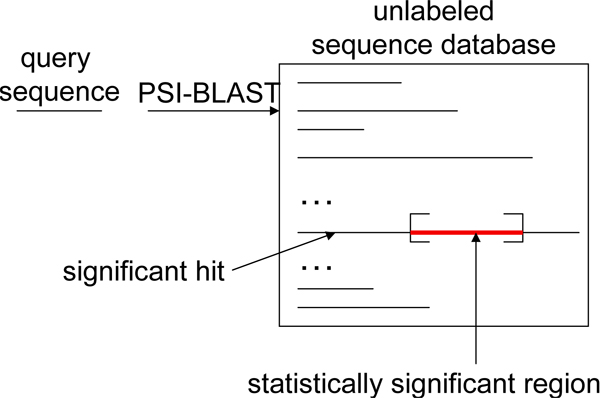
**Extracting only statistically significant regions (red/light color) from the hits**.

To establish the similarities among sequences under the semi-supervised setting, Weston et al. in [8] propose to construct the *sequence neighborhood *for each *training and testing sequence X *using the unlabeled sequences and re-represent *X *as the averaged representation of all neighboring sequences (Equation 4). The *sequence neighborhood N*(*X*) of a sequence *X *is defined as *N*(*X*) = {*X'*: *s*(*X*, *X'*) ≤ *δ*}, where *δ *is a pre-defined threshold and *s*(*X*, *X'*) is a scoring function, for example, the e-value. Under the semi-supervised learning setting, our goal is to recruit *neighbors *of training and testing sequences to construct the sequence neighborhood and use these intermediate neighbors to identify functionally or structurally related proteins that bear little to no similarity on the primary sequence level. As a result, the quality of the intermediate neighboring sequences is crucial for remote fold or homology detection. However, in many sequence databases, multi-domain protein sequences are abundant and such sequences might be similar to several unrelated single-domain sequences, as noted in [8]. Therefore, direct use of these long sequences may falsely establish similarities among unrelated sequences since these unlabeled sequences carry *excessive *and unnecessary features. In contrast, very short sequences often induce very sparse representation and therefore have *missing *features. Direct use of sequences that are too long or too short may bias the averaged neighborhood representation (4) and compromise the performance of the classifiers. Therefore, a possible remedy is to discard neighboring sequences whose lengths are substantially different from the query (training or test) sequence. For example, Weston et al. in [8] proposed to only capture neighboring sequences with maximal length of 250 (for convergence purposes). However, such practice may not offer a direct and meaningful biological interpretation. Moreover, removing neighboring sequences purely based on their length may discard sequences carrying crucial information and degrade classification performance, as we will show in Section 'Experiments'. To more effectively use unlabeled neighboring sequences, we propose to extract the *significantly similar sequence regions *from the unlabeled neighboring sequences since these regions are more likely to be *biologically relevant*. Such significant regions are commonly reported in most search methods, such as BLAST [14], PSI-BLAST [15] and HMM-based methods. We illustrate the proposed procedure using PSI-BLAST as an example in Figure 3. In the figure, given the query sequence, PSI-BLAST reports sequences (hits) containing substrings that exhibit statistically significant similarity with the query sequence. For each reported significant hit, we extract the most significant region and recruit the extracted sub-sequence as a neighbor of the query sequence. In short, the region-based neighborhood *R*(*X*) contains the *extracted significant sequence regions*, not the whole neighboring sequences of the query sequence *X*, *i.e. R*(*X*) = {*x'*: s(*X*, *X'*) ≤ *δ*}, where *x' *⊑ *X' *is the most statistically significant matching region of an unlabeled neighbor *X'*. As we will show in Section 'Experiments', the proposed region-based neighborhood method will allow us to more efficiently leverage the unlabeled data and significantly improve the classifier performance.

We summarize all competing methods for leveraging unlabeled data during training and testing under the semi-supervised learning setting in below and experimentally compare the methods in Section 'Experiments':

• *full sequence*: all neighboring sequences are recruited and the sequence neighborhood *N*(*X*) is established on the whole-sequence level. This is to show how much excessive or missing features in neighboring sequences that are too long or too short compromise the performance of the classifiers.

• *extracting the most significant region*: for each recruited neighboring sequence, we extract only the most *significantly similar sequence region *and establish the region-based neighborhood *R*(*X*) on a sub-sequence level; such sub-sequence is more likely to be biologically relevant to the query sequence.

• *filtering out long and short sequences*: for each query sequence *X*, we construct the full sequence neighborhood *N*(*X*) first (as in the full sequence method). Then we remove all neighboring sequences *X' *∈ *N*(*X*) if *T*_*X*' _> 2*T*_*X*' _or *T*_*X*' _<$\frac{T_{X}}{2}$, where *T*_*X *_is the length of sequence *X*. In essence, this method may alleviate the effect of the excessive and missing features in the full sequence method by discarding the sequences whose length fall on the tails of the length histogram.

• *maximal length of 250*: proposed by Weston et al. in [8]; for each sequence, we first construct full sequence neighborhood *N*(*X*), then we remove all neighboring sequences *X*' ∈ *N*(*X*) if *T*_*X*' _> 250.

### Clustered neighborhood kernels

The smoothing operation in Equation 4 is susceptible to overly represented neighbors in the unlabeled data set since if we append many replicated copies of a neighbor sequence to *N*(*X*), the neighbor set of *X*, the computed average will be biased towards such sequence. Large uncurated sequence databases usually contain abundant duplicated sequences. For example, some sequences in Swiss-Prot have the so-called *secondary accession numbers*. Such sequences can be easily identified and removed. However, two other types of duplication that are harder to identify are the sequences that are nearly identical and the sequences that contain substrings sharing high sequence similarity and are significant hits to the query sequence. Such sequences also may bias the estimate of the averaged representation and compromise the performance of the classifiers. Consequently, pre-processing the data prior to kernel computations is necessary to remove such bias and improve performance.

In this study we propose the *clustered neighborhood kernels*. Clustered neighborhood kernels further simplify the region neighborhood *R*(*X*) to obtain a reduced region neighborhood R*(*X*) ⊆ *R*(*X*) without duplicate or near-duplicate regions (*i.e*. with no pair of sequence regions in R*(*X*) sharing more than a pre-defined sequence identity level). The simplification is accomplished by clustering the set *R*(*X*). We then define the *clustered region-based neighborhood kernel *between two sequences *X *and *Y *as:

(6) $$K^{\prime }(X,Y)=\sum_{x\in R^{*}(X)}\sum_{y\in R^{*}(Y)}\frac{K(x,y)}{\left|R^{*}(X)||R^{*}(Y)\right|}.$$

Clustering typically incurs quadratic complexity in the number of sequences [14,16]. Moreover, *pre-clustering *the unlabeled sequence database may result in loss of neighboring sequences, which in turn may cause degradation of classifier performance, as we will discuss in Section 'Discussion on clustered neighborhood'. As a result, though clustering the union of all neighbor sets or the unlabeled dataset may appear to be more desirable, to ensure that we recruit all neighbors and to alleviate computational burden, we propose to *post-cluster *each reported neighbor set *one at a time*. For example, the union of all neighbor sets induced by the NR unlabeled database for the remote homology task contains 129, 646 sequences, while the average size of the neighbor sets is only 115. Clustering each reported neighbor set individually leads to significant savings in running time, especially when coupled with kernel methods that are computationally expensive, as we will illustrate experimentally in Section 'Discussion on clustered neighborhood'.

### Experiments

**Figure 4:**
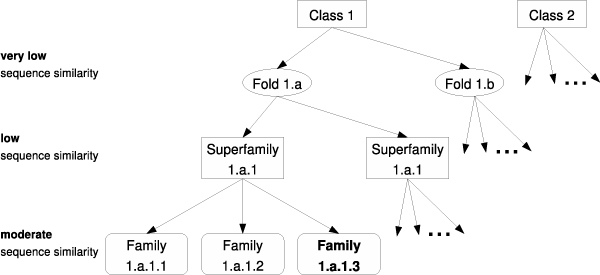
**The SCOP (Structural Classification of Proteins) hierarchy**.

We perform the remote fold and remote homology detection experiments under the SCOP [17] (Structural Classification of Proteins) classification. Proteins in the SCOP dataset are placed in a tree hierarchy: class, fold, superfamily and family, from root to leaf as illustrated in Figure 4. Proteins in the same superfamily are very likely to be evolutionarily related; on the other hand, proteins in the same fold share structural similarity but are not necessarily homologous. For *remote homology detection *under the semi-supervised setting we use the standard SCOP 1.59 data set, published in [8]. The data set contains 54 *binary *classification problems, each simulating the remote homology detection problem by training on a subset of families under the target superfamily and testing the superfamily classifier on the remaining (held out) families. For the *remote fold prediction *task we use the standard SCOP 1.65 data set from [12]. The data set contains 26 folds (26-way *multi-class *classification problem), 303 superfamilies and 652 families for training with 46 superfamilies completely held out for testing to simulate the *remote *fold recognition setting.

To perform experiments under the *semi-supervised *setting, we use three unlabeled sequence databases, some containing abundant multi-domain protein sequences and duplicated or overly represented (sub-)sequences. The three databases are PDB [18] (as of Dec. 2007, 17,232 sequences), Swiss-Prot [19] (we use the same version as the one used in [8] for comparative analysis of performance; 101,602 sequences), and the *non-redundant *(NR) sequence database (534,936 sequences). To adhere to the true semi-supervised setting, we remove *all sequences in the unlabeled data sets identical to any test sequences*.

To construct the sequence neighborhood of *X*, we perform two PSI-BLAST iterations on the unlabeled database with *X *as the query sequence and recruit all sequences with e-values ≤ .05. These sequences now form the neighborhood *N*(*X*) at the full sequence level. Next for each neighboring sequence, we extract the most significant region (lowest e-value) to form the sub-sequence (region) neighborhood *R*(*X*). Finally, we cluster *R*(*X*) at 70% sequence identity level using an existing package, *cd-hit *[16], and form the clustered region neighborhood R*(*X*) using the representatives. The region-based neighborhood kernel then can be obtained using the smoothed representations (Equation 4) by substituting *N*(*X*) with *R*(*X*) or R*(*X*). We evaluate our methods using the spatial sample and the mismatch representations (Sections 'The spectrum kernel family' and 'The Sparse Spatial Sample Features').

In all experiments, we normalize the kernel values *K*(*X*, *Y*) using $K^{\prime }(X,Y)=\frac{K(X,Y)}{\sqrt{K(X,X)K(Y,Y)}}$ to remove the dependency between the kernel value and the sequence length. We use sequence neighborhood smoothing in Equation 4, as in [8], under both the spatial sample and mismatch representations. To perform our experiments, we use an existing SVM implementation from a standard machine learning package SPIDER [20] with default parameters.

For the sparse spatial sample kernel, we use triple(1,3) (*k *= 1, *t *= 3 and *d *= 3), *i.e*. features are *triples *of monomers, and for the mismatch kernel, we use mismatch(5,1) (*k *= 5, and *m *= 1) and mismatch(5,2) kernels. To facilitate large-scale experiments with relaxed mismatch constraints and large unlabeled datasets, we use the algorithms proposed by Kuksa et al. in [21].

For the *remote homology *(superfamily) detection task, we evaluate all methods using the *Receiver Operating Characteristic *(ROC) and ROC50 [22] scores. The ROC50 score is the (normalized) area under the ROC curve computed for up to 50 false positives. With a small number of positive test sequences and a large number of negative test sequences, the ROC50 score is typically more indicative of the prediction accuracy of a homology detection method. Higher ROC/ROC50 scores suggest better discriminative power of the classifier.

For the *remote fold recognition *task, we adopt the standard proposed by Melvin et al. in [12] and use 0–1 and balanced error rates as well as the F1 scores (*F*1 = 2*pr*/(*p *+ *r*), where *p *is the precision and *r *is the recall) to evaluate the performance of the methods (lower error rates and/or higher F1 scores suggest better discriminative power of the multi-class classifier). Unlike the remote homology (superfamily) detection task, which was formulated as a *binary *classification problem, the remote fold detection task was formulated as a *multi-class *classification problem; currently, there is no clear way of evaluating such classification problem using the ROC scores. Data and source code are available at the supplementary website [23].

### Remote homology (superfamily) detection experiments

In this section, we compare the results obtained using region-based and full sequence methods on the task of *superfamily (remote homology) detection*. We first present the results obtained using the spatial SSSK kernels (Section 'The Sparse Spatial Sample Features').

#### Experimental results with the triple(1,3) kernel

**Figure 5:**
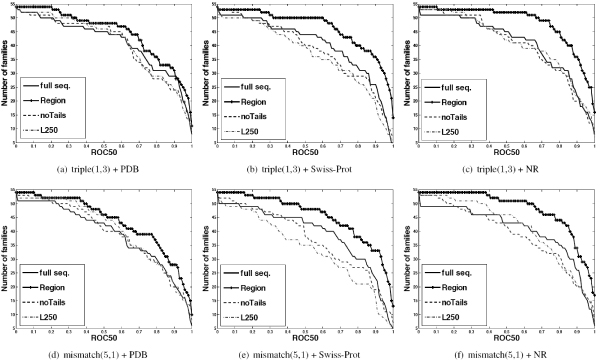
**ROC50 plots of four competing methods using the triple-(1,3) and mismatch-(5,1) kernels with PDB, Swiss-Prot and NR as unlabeled databases for remote homology prediction**.

In the upper panel of Figure 5, we show the ROC50 plots of all four competing methods, *with post-clustering*, using the triple(1,3) kernel on different unlabeled sequence databases (PDB, Swiss-Prot, and NR). In each figure, the horizontal axis corresponds to a ROC50 score, and the vertical axis denotes the number of experiments, out of 54, with equal or higher ROC50 score (an ideal method will result in a horizontal line with *y*-coordinate corresponding to the total number of experiments). In all cases, we observe the ROC50 curves of the *region-based *method (lines with '+' signs) show strong dominance over those of other methods that use full sequences. Furthermore, as we observe in Figures 5(a) and 5(b), discarding sequences based on the sequence length (the two colored dashed and dashed-dotted lines) degrades the performance of the classifiers compared to the baseline (full sequence) method (solid lines). This suggests that longer unlabeled sequences carrying crucial information for inferring the class labels of the test sequences are discarded.

We summarize performance measures (average ROC and ROC50 scores) for all competing methods in Table 1 (with and without post-clustering). For each method, we also report the p-value of the Wilcoxon Signed-Rank test on the ROC50 scores against the *full sequence *(baseline) method. The *region-based *method strongly outperforms other competing methods that use full sequences and consistently shows statistically significant improvements over the baseline full-sequence method, while the other two methods suggest no strong evidence of improvement. We also note that clustering significantly improves the performance of the full sequence method (p-value < .05 in all unlabeled datasets) and offers noticeable improvements for the region-based method on larger datasets (*e.g*. NR). Clustering also results in substantial reduction in running times, as we will show in Section 'Discussion on clustered neighborhood'.

**Table 1:** Experimental results on the remote homology detection task for all competing methods using the triple(1,3) kernel.

	neighborhood (no clustering)			clustered neighborhood		
dataset	ROC	ROC50	p-value	ROC	ROC50	p-value
PDB						

full sequence	.9476	.7582	-	.9515	.7633	-
region	.9708	**.8265**	**.0069**	.9716	**.8246**	**.0045**
no tails (full seq.)	.9443	.7522	.5401	.9472	.7559	.5324
max length (full seq.)	.9471	.7497	.4407	.9536	.7584	.5468

Swiss-Prot						

full sequence	.9245	.6908	-	.9464	.7474	-
region	.9752	**.8556**	**2.46e-04**	.9732	**.8605**	**1.5e-03**
no tails (full seq.)	.9361	.6938	.8621	.9395	.7160	.6259
max length (full seq.)	.9300	.6514	.2589	.9348	.6817	.1369

NR						

full sequence	.9419	.7328	-	.9556	.7566	-
region	.9824	**.8861**	**1.08e-05**	.9861	**.8944**	**2.2e-05**
no tails (full seq.)	.9575	.7438	.6640	.9602	.7486	.8507
max length (full seq.)	.9513	.7401	.8656	.9528	.7595	.8696

* p-value: signed-rank test on ROC50 scores against* full sequence *in the corresponding setting

#### Experimental results on remote homology detection with the mismatch(5,1) kernel

In the lower panel of Figure 5, we show the ROC plots of all four competing methods, *with post-clustering*, using the mismatch(5,1) kernel on different unlabeled sequence databases (PDB, Swiss-Prot, NR). We observe that the ROC50 curves of the *region-based *method show strong dominance over those of other competing methods that use full sequences. In Figures 5(e) and 5(f) we again observe the effect of filtering out unlabeled sequences based on the sequence length: longer unlabeled sequences carrying crucial information for inferring the label of the test sequences are discarded and therefore the performance of the classifiers is compromised. Table 2 compares performance of region-based and full-sequence methods using mismatch(5,1) kernel (with and without post-clustering) on the remote homology task. The *region-based *method again shows statistically significant improvement compared to the full sequence and other methods. Interestingly, using Swiss-Prot as an unlabeled database, we observe that filtering out the sequences with length > 250 *degrades *the performance significantly. Similar to the triple kernel, we also observe significant improvements for the *full sequence *method with clustered neighborhood on larger datasets.

**Table 2:** Experimental results for all competing methods on the remote homology detection task using the mismatch(5,1) kernel.

	neighborhood (no clustering)			clustered neighborhood		
dataset	ROC	ROC50	p-value	ROC	ROC50	p-value
PDB						

full sequence	.9389	.7203	-	.9414	.7230	-
region	.9698	**.8048**	**.0075**	.9705	**.8038**	**.0020**
no tails (full seq.)	.9379	.7287	.9390	.9378	.7301	.7605
max length (full seq.)	.9457	.7359	.4725	.9526	.7491	.3817

Swiss-Prot						

full sequence	.9253	.6685	-	.9378	.7258	-
region	.9757	**.8280**	**.0060**	.9773	**.8414**	**.0108**
no tails (full seq.)	.9290	.6750	.9813	.9344	.6874	.5600
max length (full seq.)	.9185	.6094	.1436	.9223	.6201	.0279

NR						

full sequence	.9475	.7233	-	.9544	.7510	-
region	.9837	**.8824**	**1.7e-04**	.9874	**.8885**	**1.2e-04**
no tails (full seq.)	.9554	.7083	.7930	.9584	.7211	.7501
max length (full seq.)	.9508	.7421	.7578	.9518	.7613	.9387

* p-value: signed-rank test on ROC50 scores against* full sequence *in the corresponding setting

### Multi-class remote fold recognition experiments

In the remote fold recognition setting, the classifiers are trained on a number of superfamilies under the fold of interest and tested on unseen superfamilies. The task is also made harder by switching from the *binary *setting in the remote homology task in Section 'Remote homology (superfamily) detection experiments' to the *multi-class *setting. We adopt the simple one-vs-all scheme used by Kuksa et al. in [24]: let *Y *be the output space, we estimate |*Y*| binary classifiers and given a sequence *x *we predict the class $\hat{y}$ using equation 7, where *f*_*y *_denotes the classifier built for class *y *∈ *Y*.

(7) $$\hat{y}=\underset{y\in Y}{\arg\max}f_{y}(x),$$

In Table 3 we compare the classification performance (0–1 and balanced error rates as well as F1 scores) on the multi-class remote fold recognition task of the region-based and the full-sequence methods using the triple(1,3) kernel with post-clustering. Under the top-*n *error cost function, a classification is considered correct if *f*_*y*_(*x*) has rank, obtained by sorting all prediction confidences in non-increasing order, at most *n *and *y *is the true class of *x*. On the other hand, under the balanced error cost function, the penalty of mis-classifying one sequence is inversely proportional to the number of test sequences in the target class (i.e. mis-classifying a sequence from a class with a small number of examples results in a higher penalty compared to that of mis-classifying a sequence from a large, well represented class). From the table we observe that in all instances, the region-based method demonstrates significant improvement over the baseline (full sequence) method (e.g. top-1 error reduces from 50.8% to 36.8% by using regions) whereas filtering sequences based on the length show either no clear improvement or noticeable degradation in performance.

**Table 3:** Multi-class remote fold recognition using the triple(1,3) kernel

Method	Error	Top-5 Error	Balanced Error	Top-5 Balanced Error	F1	Top-5 F1
full sequence	50.81	17.92	71.95	27.80	28.92	73.93
region	**36.81**	**10.91**	**52.58**	**20.07**	**49.69**	**81.26**
no tails (full seq.)	48.21	19.71	70.42	33.37	30.91	73.39
max. length (full seq.)	51.63	23.13	76.96	39.21	26.85	66.99

Table 4 summarizes the performance measures for all competing methods on multi-class remote fold prediction task using the mismatch(5,1) kernel with post-clustering. We again observe that region-based methods clearly outperform all other competing methods (e.g. top-1 error reduces from 50.5% to 44.8% using regions).

**Table 4:** Multi-class remote fold recognition performance using the mismatch(5,1) kernel

Method	Error	Top-5 Error	Balanced Error	Top-5 Balanced Error	F1	Top-5 F1
full sequence	50.49	22.31	76.44	38.61	24.96	65.58
region	**44.79**	**13.36**	**67.26**	**25.40**	**33.17**	**77.45**
no tails (full seq.)	51.79	20.85	79.66	35.72	22.72	66.68
max. length (full seq.)	56.03	26.06	86.68	47.05	15.04	58.36

In Table 5, we compare the performance of all competing methods with and without clustering, using the mismatch(5,2) similarity measure for the remote fold recognition task (we use relaxed matching [21] (*m *= 2) since mismatch(5,1) measure is too stringent to evaluate similarity in the case of very low sequence identities at the fold level). As we can see from Table 5, relaxed matching for the mismatch kernel (*m *= 2) further improves accuracy (compare with Table 4) with region-based method (e.g. region-based method results in a top-1 error of 40.88% compared to 50.16% of the baseline). Sequence neighborhood clustering also substantially improves the classification accuracy in most of the cases.

**Table 5:** Multi-class remote fold recognition using the mismatch(5,2) kernel

Method	Error	Top-5 Error	Balanced Error	Top-5 Balanced Error	F1	Top-5 F1
Without clustering						

full seq.	50.16	21.82	67.17	32.55	37.43	71.40
region	42.83	13.68	61.43	**22.63**	40.36	**79.19**
no tails (full seq.)	50.16	21.82	71.81	32.59	30.17	69.12
max. length (full seq.)	52.44	24.43	77.31	39.17	23.98	65.22

With clustering						

full seq.	50.33	19.71	70.04	27.21	32.10	75.03
region	**40.88**	**13.68**	**57.86**	22.82	**47.54**	79.03
no tails (full seq.)	48.37	20.68	69.83	32.27	31.48	70.03
max. length (full seq.)	52.44	23.29	77.05	36.52	26.84	68.02

### Comparison with other state-of-the-art methods

In Table 6, we compare *remote homology detection *performance our proposed methods on two string kernels (triple and mismatch) against the profile kernel, the state-of-the-art method for remote homology (superfamily) detection. We use the code provided in [10] to construct the profile kernels. We also control the experiments by strictly adhering to the semi-supervised setting to avoid giving advantage to any method. For each unlabeled data set, we highlight the methods with the best ROC and ROC50 scores. In almost all cases, the *region-based *method with clustered neighborhood demonstrates the best performance. Moreover, the ROC50 scores of the triple and mismatch kernels strongly outperform those of the profile kernel. We note that previous studies [7,8] suggest that the profile kernel outperforms the mismatch neighborhood kernel. However, we want to point out that the profile kernel constructs profiles using smaller matching segments, not the whole sequence. Therefore, a direct comparison between profile and the original neighborhood mismatch kernels [8] may give the profile kernel a slight advantage, as we have clearly shown by the full sequence (whole sequence) method in Section 'Experimental results on remote homology detection with the mismatch(5,1) kernel'. Previous results for the mismatch neighborhood kernels, though promising, show a substantial performance gap when compared to those of the profile kernels. Moreover, as shown in [7], to improve the accuracy of the profile kernels, one needs to increase the computationally demanding PSI-BLAST iterations. Using the *region-based neighborhood *with only 2 PSI-BLAST iterations both mismatch and spatial neighborhood kernels achieve results better than profile kernels with 5 PSI-BLAST iterations [7]. In this study, we bridge the performance gap between the profile and mismatch neighborhood kernels and show that by establishing the sub-sequence (region) neighborhood, the mismatch neighborhood kernel outperforms the profile kernel.

**Table 6:** Comparison of performance against the state-of-the-art methods for remote homology detection

	PDB		Swiss-Prot		NR	
	
	ROC	ROC50	ROC	ROC50	ROC	ROC50
triple(1,3), full seq.	.9475	.7582	.9245	.6908	.9419	.7327
triple(1,3), region	.9708	**.8265**	.9752	.8556	.9824	.8861
triple(1,3), region, clustering	**.9716**	.8246	.9732	**.8605**	.9861	**.8944**
mismatch(5,1), full seq.	.9389	.7203	.9253	.6685	.9423	.7233
mismatch(5,1), region	.9698	.8048	.9757	.8280	.9837	.8824
mismatch(5,1), region, clustering	.9705	.8038	**.9773**	.8414	**.9874**	.8885
profile(5,7.5)	.9511	.7205	.9709	.7914	.9734	.8151

In Table 7, we compare our proposed methods for *multi-class remote fold recognition *using two string kernels (triple and mismatch) against the state-of-the-art profile kernel method. All semi-supervised learning methods are accomplished with 2 PSI-BLAST iterations using non-redundant unlabeled data set (NR); all sequences that are identical to any test sequences are removed. We again observe that region-based method, especially when coupled with the spatial (triple) kernel, significantly outperform the profile kernel.

**Table 7:** Comparison with the state-of-the-art methods for multi-class remote fold recognition

Method	Error	Top-5 Error	Balanced Error	Top-5 Balanced Error	F1	Top-5 F1
mismatch (full seq.)	50.49	22.31	76.44	38.61	24.96	65.58
triple (full seq.)	50.81	17.92	71.95	27.80	28.92	73.93
mismatch (region)	44.79	13.36	67.26	25.40	33.17	77.45
triple (region)	**36.81**	**10.91**	**52.58**	**20.07**	**49.69**	**81.26**
profile(5,7.5)	45.11	15.80	71.27	31.55	32.34	75.68
profile(5,7.5)†	46.30	14.50	62.80	23.50	-	-

†: directly quoted from [12]

**Figure 6:**
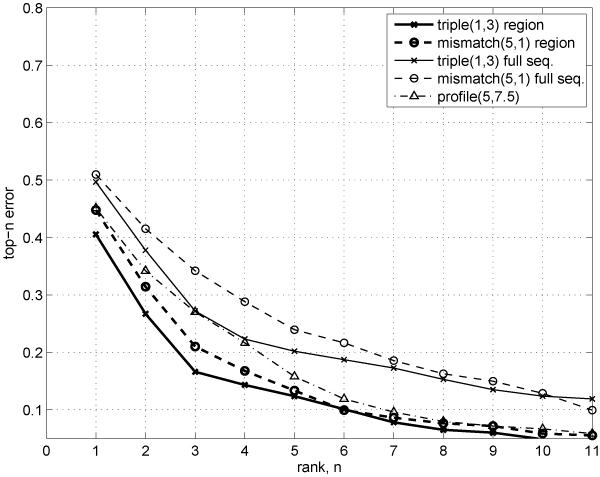
**Ranking quality (0–1 top-*n *error rates) for the multi-class remote fold recognition task under the semi-supervised setting**.

**Figure 7:**
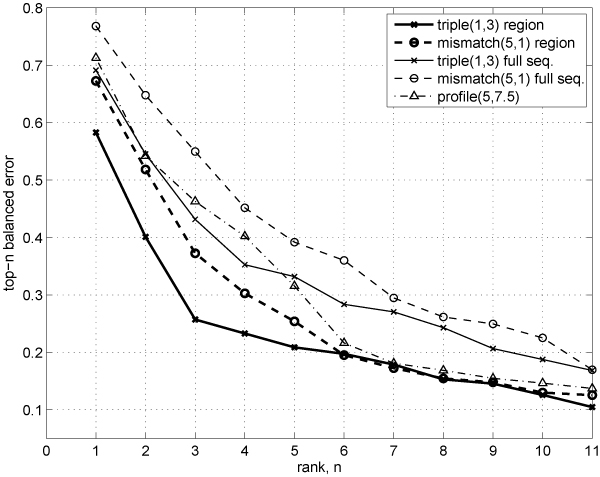
**Ranking quality (top-*n balanced *error rates) for the multi-class remote fold recognition under semi-supervised setting**.

In Figures 6 and 7, we compare ranking quality on the *multi-class remote fold recognition *task for region-based and full sequence-based methods using the 0–1 top-*n *error and the top-*n *balanced error curves. The region-based methods clearly show strong dominance in ranking quality over the baseline (full sequence) methods and the profile kernel for small values of *n*.

## Discussion

We further discuss the benefits of extracting only statistically significant regions from the neighboring sequences in Section 'Motivation for region extraction' and elaborate on the role of post-clustering in Section 'Discussion on clustered neighborhood'.

### Motivation for region extraction

**Figure 8:**
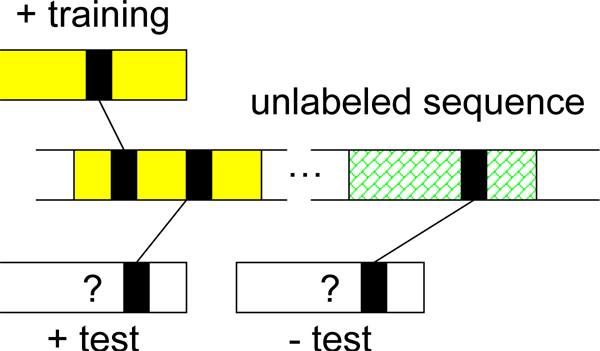
**The importance of only extracting relevant region from neighboring sequences (middle) for inferring sequence labels**.

Figure 8 illustrates the benefit of extracting only statistically significant regions from the unlabeled sequences. In the figure, colors indicate membership: yellow (shaded) represents the positive class and green (pattern) the negative class. The arcs indicate (possibly weak) similarity induced by shared features (black boxes) and absence of arcs indicates no similarity. Sequences sharing statistically significant similarity are more likely to be evolutionarily/structurally related and therefore to belong to the same superfamily/fold. The goal is to infer membership of the test (unshaded) sequences via the unlabeled sequence (middle). As can be seen from the figure, the positive training and test sequences share no features and therefore no similarity; however, the unlabeled sequence shares some features with both sequences in the reported region, which is very likely to be biologically or structurally related to both positive sequences. Via this unlabeled sequence, the similarity between the two positive sequences is established. In contrast, if the whole unlabeled sequence is recruited as a neighbor, the similarity between the positive training and negative test sequences will be falsely established by the irrelevant regions, resulting in poor classifier performance.

One example in the SCOP 1.59 dataset that demonstrates this behavior is the target family *EGF-type module *under the *EGF/Laminin *superfamily, *Knottins *fold and *small proteins *class. In the experiment, we observe an unlabeled sequence in Swiss-Prot (ID Q62059) sharing statistically significant similarity to the positive training, positive test, and negative test sequences. The class and fold pairs observed in similar negative test sequences are (*all beta*, *Immunoglobulin-like beta sandwich*), (*alpha+beta*, *C-type lectin-like*), and (*small proteins*, *complement control module/SCR domain*). Swiss-Prot annotation states that this protein sequences contain the *C-type lectin*, *Immunoglobulin-like V-type*, *link *and *sushi (CCP/SCR) *domains. Without region extraction, the ROC50 scores are 0.3250 and 0.3292 under the triple and mismatch kernels. By establishing the neighborhood based on the extracted regions, the ROC50 scores improve to 0.9464 and 0.9664.

### Discussion on clustered neighborhood

In Section 'Clustered Neighborhood Kernels', we propose to *post-cluster *each sequences neighbor set *one at a time*, as opposed to *pre-clustering *the union of all neighbor sets or the whole unlabeled sequence database. In this section, we further illustrate the benefits of post-clustering: improvement in performance of classifiers as well as reduced storage and running time for classification.

We first show the difference between pre- and post-clustering using the PDB database under the remote homology detection task. For *pre-clustering*, we cluster the whole PDB database at 70% sequence identity level to obtain PDB70. Then we perform 2 PSI-BLAST iterations on PDB70 to obtain the sequence neighborhood and extract the significant regions. In contrast, for *post-clustering*, we perform 2 PSI-BLAST iterations on the whole PDB database, extract the significant regions for each neighboring sequence and then cluster the extracted regions at 70% sequence identity level. For the triple(1,3) neighborhood kernel, the ROC-50 scores for pre-/post- clustering are .8122 and .8246 with a border-line significant p-value of .1248. For the mismatch(5,1) kernel, the ROC-50 scores for pre-/post-clustering are .7836 and .8038 with a significant p-value of .0853. Under the pre-clustering framework, the mean/median/max number of neighbors for each labeled sequence is 11/4/119 whereas under the post-clustering framework, the number of neighbors is 11/5/130; performing post-clustering in general slightly increases the number of neighbors for each labeled sequence. In fact, under the post-clustering framework, we scan the whole unlabeled sequence database to find the neighbors of a query sequence and recruit *all *neighboring sequences. Furthermore, during the later clustering stage, a neighboring sequence will be removed only if there is another similar sequence in the neighborhood, whereas under the pre-clustering framework, when a potential neighbor is removed and a representative chosen for the corresponding cluster, the representative might be too dissimilar to the query sequence and might not be recruited as a neighbor, which might result in worse performance as shown on PDB database. In addition to improving classification accuracy, performing clustering on the neighbor sets may also lead to substantial reduction in storage space and computational time. Our experimental data shown in Table 8 suggests that performing clustering reduces the neighborhood size by two fold on average, which in turn implies less computational resources for storage: under the discriminative kernel learning setting, we need to save the support vectors along with their corresponding neighbor sets. In Table 9, we show the experimental running time, in seconds, for computing the 3,860 × 3,860 mismatch and triple kernel matrices for the fold recognition task. By extracting the significant regions of the neighboring sequences, the experimental running time has been reduced substantially compared to full sequence-based methods. Performing clustering on a per sequence neighborhood basis further reduces running time. The neighborhood size as well as the number of features also reduces substantially by using regions and post-clustering, as illustrated in Tables 9 and 8.

**Table 8:** The number of neighbors (mean/median/maximum) and the number of observed features with and without clustering for the remote fold recognition task

Method	Without Clustering		With Clustering	
	# neighbors	# features	# neighbors	# features
full seq.	135/99/490	192,378,952	64/41/356	120,990,413
region	64/41/356	34,807,209	50/26/352	28,738,521
no tails (full seq.)	75/17/402	57,575,176	23/11/325	29,649,870
max. length (full seq.)	70/16/431	39,915,003	22/12/279	14,634,511

**Table 9:** Running time for kernel matrix computation (3860 × 3860), [s]

Method	mismatch(5,1)	mismatch(5,2)	triple(1,3)
full seq.	12,084	13,593	153
region	2,624	3,195	73
region+clustering	2,412	2,998	64

## Conclusion

We propose a systematic and biologically motivated computational approach for extracting relevant information from unlabeled sequence databases for the task of primary protein sequence classification using sequence kernels. We also propose the use of the clustered neighborhood kernels to improve the classifier performance and remove the kernel estimation bias caused by overly-represented sequences in large uncurated databases. Combined with two state-of-the-art string kernels (spatial and mismatch), our framework significantly improves accuracy and achieves the state-of-the-art prediction performance on semi-supervised protein remote fold recognition and remote homology detection. The improvements in performance accuracy are matched with significantly reduced computational running times. Just as one would need to cut and polish a gemstone to bring out its beauty, to take full advantage of the unlabeled neighboring sequences, one also needs to carefully extract only relevant regions that are more likely to be biologically or structurally related. The unlabeled sequences here resemble the unpolished gemstones; when used as-is, they may carry unnecessary features and hence compromise the classification accuracy but once cut and polished, they improve the accuracy of the classifiers considerably. Our approach can be directly extended to other challenging analysis tasks, such as clustering, functional prediction, or localization of protein sequences.

## Competing interests

The authors declare that they have no competing interests.

## Authors' contributions

All authors contributed equally to this publication.
